# Development of novel osteoarthritis therapy by targeting AMPK-β-catenin-Runx2 signaling

**DOI:** 10.1016/j.gendis.2024.101247

**Published:** 2024-02-24

**Authors:** Daofu Zeng, Muhammad Umar, Zhenglin Zhu, Haobo Pan, William W. Lu, Guozhi Xiao, Yan Chen, Liping Tong, Di Chen

**Affiliations:** a Department of Bone and Joint Surgery, The First Affiliated Hospital of Guangxi Medical University, Nanning, Guangxi 530021, China; b Research Center for Computer-aided Drug Discovery, Shenzhen Institute of Advanced Technology, Chinese Academy of Sciences, Shenzhen, Guangdong 518055, China; c Faculty of Pharmaceutical Sciences, Shenzhen Institute of Advanced Technology, Shenzhen, Guangdong 518055, China; d Department of Orthopedic Surgery, The First Affiliated Hospital of Chongqing Medical University, Chongqing 400016, China; e Shenzhen Healthemes Biotechnology Co., Ltd., Shenzhen, Guangdong 518071, China; f School of Medicine, Southern University of Science and Technology, Shenzhen, Guangdong 518055, China

**Keywords:** β-catenin, AMPK, Osteoarthritis, Runx2, Signaling pathway

## Abstract

Osteoarthritis (OA) is a debilitating chronic joint disease affecting large populations of patients, especially the elderly. The pathological mechanisms of OA are currently unknown. Multiple risk factors are involved in OA development. Among these risk factors, alterations of mechanical loading in the joint leading to changes in biological signaling pathways have been known as a key event in OA development. The importance of AMPK-β-catenin-Runx2 signaling in the initiation and progression of OA has been recognized in recent years. In this review, we discuss the recent progress in understanding the role of this signaling pathway and the underlying interaction mechanisms during OA development. We also discuss the drug development aiming to target this signaling pathway for OA treatment.

## Introduction

**Figure 1:**
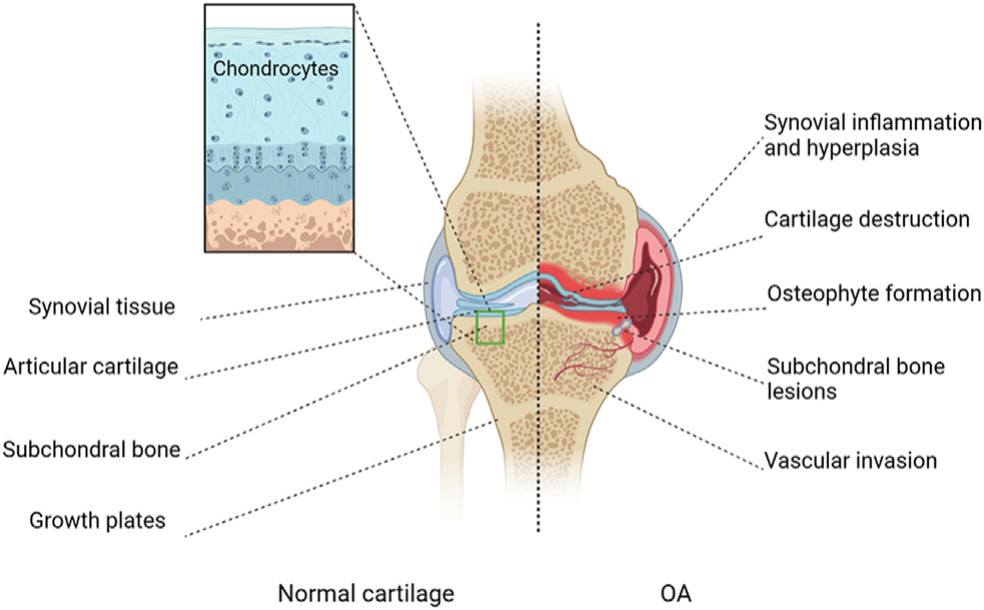
Schematic diagram of normal articular cartilage and OA cartilage. OA is a disease involving the whole joint, and its pathological changes include cartilage erosion, synovial inflammation and hyperplasia, osteophyte formation, abnormal vascular invasion, subchondral bone disorder, and reconstruction. The left side is normal articular cartilage tissue and cartilage local enlargement, and the right side is OA cartilage tissue. OA, osteoarthritis.

Osteoarthritis (OA) is a common and serious joint disease. Its primary clinical symptoms are pain and deformity, and severe cases can lead to disability.^1^ The pathological changes of OA are wide-ranging from cartilage erosion, synovial inflammation, subchondral bone sclerosis, to osteophyte formation (Fig. 1).^1^ Currently, the treatment methods for OA mainly include physical therapy, drug intervention, and surgery.^2^ The primary purpose of these approaches is to control pain symptoms, reduce or slow down joint damage, and reduce disability.^2^ However, it cannot reverse the pathological changes of OA and prevent the progression of OA. When OA progresses to an advanced stage, artificial joint replacement is the only option for OA patients.^3^ How to treat OA, reduce pain in patients with OA, and slow the progression of OA still needs more research. Recent signaling studies help us understand pathological mechanisms of OA initiation and progression. Several signaling pathways were found to be involved in the development of OA, including fibroblast growth factor, transforming growth factor-β/bone morphogenetic protein, nuclear factor-κB, and hypoxia-inducible factor.^4^ Further research on the associated pathogenic signaling pathways will contribute to a deeper and comprehensive understanding of the pathogenesis of OA, and drugs targeting these signaling pathways may hold potential for OA treatments.

The pathogenesis of OA is complex, involving changes in various cell signals, and its specific pathogenesis has yet to be fully understood. AMP-activated protein kinase (AMPK), β-catenin, and Runt-related transcription factor 2 (Runx2) are key molecules or molecular targets of cell signaling and play an essential role in the development of organisms. Changes in these key molecules are related to the occurrence and development of diseases.^5^, ^6^, ^7^ With the research in human genetics and experimental animal models, it has been known that alterations in AMPK, β-catenin, and Runx2 are the key events that lead to the emergence and progression of OA, contributing to the pathogeny of OA.^4^ Up-regulation or inhibition of the expression of these signaling molecules or alterations of the molecular interactions within the AMPK-β-catenin-Runx2 signaling pathway will affect the pathological consequence of OA. The development of drugs targeting these molecules will be an effective way to exploit the prevention and treatment of OA. In this writing, we will focus on the recent progress in understanding the effects and mechanisms of these signaling molecules and their interactions during OA development. We will summarize the drug development aiming at targeting this signaling pathway for OA treatment. Finally, we reviewed the latest research advances in treating OA using natural products.

## AMPK-β-catenin-Runx2 signaling in OA

### AMPK

AMPK is the main energy-sensing enzyme in mammalian cells and plays a pivotal role in cell metabolism and systemic energy balance. AMPK is a heterotrimeric complex consisting of a catalytic α subunit, two regulatory β subunits, and an γ subunit.^8^ The α and β subunits have two isoforms, while the γ subunit has three isoforms.^9^^,^^10^ Twelve different AMPK molecules can be a combination of different subunits, which are differentially expressed and play different roles in various cell types in mammals.^9^^,^^10^ AMPK is a highly conserved enzyme that senses energy status and regulates cell metabolism and energy homeostasis by sensing changes in intracellular levels of energy molecules such as AMP, ADP, and ATP.^11^ The changes in ATP and AMP levels in cells are the main factors mediating the activation of AMPK.^11^ When the energy metabolism in the body is out of balance, such as an increase in AMP levels or a decrease in ATP content, the AMPK activity can be activated through a variety of pathways: i) The interaction of AMP with AMPK leads to allosteric modification of AMPK, which weakens the dephosphorylation of Thr172 by phosphatase^12^; ii) The combination of AMP and AMPK will phosphorylates AMPK at Thr172 by promoting the expression of its upstream proteases including liver kinase B1, transforming growth factor-β-activated protein kinase-1, and calcium-sensitive kinase CaMKK2^13^^,^^14^; iii) A high ratio of AMP/ATP will reduce the dephosphorylation of AMPK by protein phosphatase, thus increasing the phosphorylation level of AMPK.^15^^,^^16^ Activated AMPK reduces ATP consumption by inhibiting anabolic processes while promoting catabolic processes and increasing ATP production to restore energy balance.^17^

**Figure 2:**
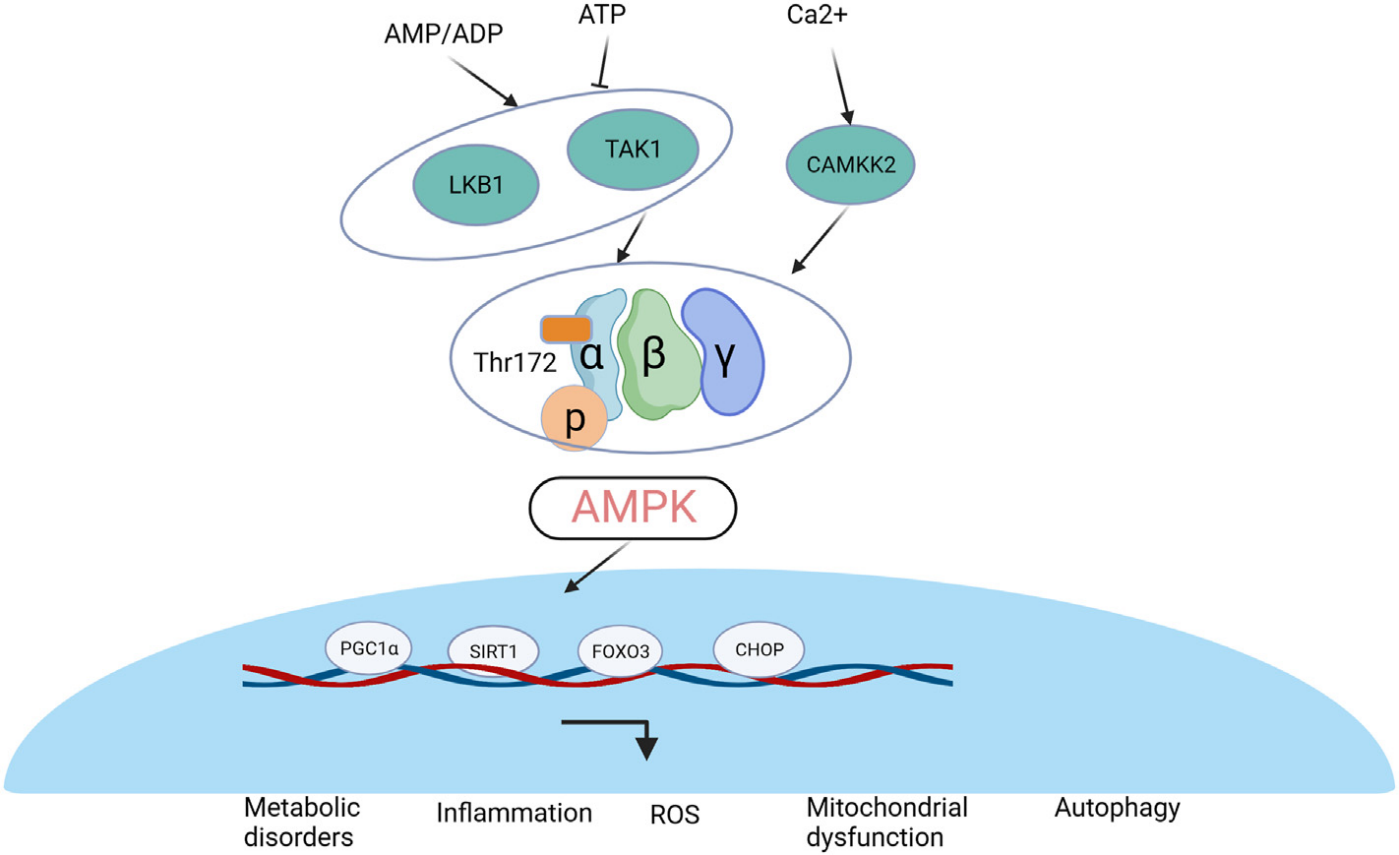
AMPK signaling in OA chondrocytes. AMPK is an important energy receptor with three subunits (α, β, and γ). LKB1, TAK1, CAMKK2 are key molecules that promote phosphorylation of AMPK, regulated by AMP/ADP, ATP, and Ca^2+^. The activity of AMPK in OA chondrocytes decreases, regulating the expression of its downstream target genes such as PGC1α, SIRT1, FOXO3, and CHOP, which ultimately leads to chondrocyte metabolic disorders, inflammatory response, oxidative stress, mitochondrial dysfunction, and chondrocyte autophagy. LKB1, liver kinase B1; TAK1, transforming growth factor-β-activated protein kinase-1; CAMKK2, calcium/calmodulin-dependent kinase; AMPK, AMP-activated protein kinase; PGC1α, peroxisome proliferator–activated receptor γ coactivator 1α; SIRT1, sirtuin 1; FOXO3, forkhead box O3; CHOP, C/EBP homologous protein; ROS, reactive oxygen species.

Existing research findings have shown that AMPK plays a vital role in the process of anabolism and catabolism *in vivo* and mediates in the regulation of energy balance. Altered AMPK levels are in reference to the development of several diseases, such as neurodegenerative diseases, diabetes, cardiovascular disease, obesity, and OA.^18^, ^19^, ^20^ Cumulative evidence shows that low-grade inflammation caused by metabolic disorders of chondrocytes can promote the occurrence and progression of OA.^21^ AMPK is widely expressed in chondrocytes, and as a significant molecule in the regulation of cell metabolic activity, it may play a very important role in the advent and development of OA (Fig. 2). Studies on human OA chondrocytes showed that AMPK activity was inhibited in OA chondrocytes, similarly, AMPK also showed reduced activity in chondrocytes of OA mouse models.^22^^,^^23^ In addition, decreased AMPKα phosphorylation was also observed in interleukin-1β and tumor necrosis factor-alpha-induced chondrocytes, and inhibition of AMPK activity could further enhance the catabolic response of inflammatory factors to chondrocytes.^22^^,^^24^ These findings demonstrate an integral role of AMPK activity in the homeostasis of articular chondrocytes, and the energy disorder caused by the decrease of AMPK activity may be one of the key events leading to joint inflammation.

In a tamoxifen-inducible chondrocyte-specific AMPK knockout mouse model, it was found that the loss of AMPK expression increased the severity of OA in mice in knee joint unstable OA mouse model.^25^ The cartilage destruction in AMPK knockout mice was more severe, and higher expression levels of matrix metalloproteinases (MMPs) and increased cellular apoptosis were detected in articular cartilage.^25^ Furthermore, increased synovial inflammation was observed in aged AMPK knockout mice.^25^ Therefore, the lack of AMPK may be related to age-related OA, but further investigation is needed to verify reported findings because critical and reliable controls are needed in this type of study.

Proper energy metabolism is important for cells to maintain normal functions. The disturbance of energy balance will lead to the imbalance of homeostasis and functional damage in the cell, thereby involving in the disease development. AMPK signaling has been shown to be involved in OA.^21^ Dysfunction of AMPK signaling in chondrocytes leads to cellular dysfunction by inducing a series of cellular stress responses, such as mitochondrial dysfunction, oxidative stress, and inflammation, ultimately promoting OA development and progression.^26^^,^^27^ Given the important roles of AMPK in energy metabolism and OA, targeting AMPK signaling may be a potential approach for treating OA and preventing its progression.

### β-catenin

β-catenin is an evolutionarily conserved molecule that plays a crucial role in the development and homeostasis of living organisms. β-catenin is not only a key molecule to regulate cell adhesion, but also a core factor to promote the activation of canonical Wnt signaling in the nucleus, mediating a variety of intercellular signal transductions.^28^ It is known that β-catenin protein in the cell could interact with the cytoplasmic domain cadherin and participate in the adhesion between cells.^28^^,^^29^ The Wnt signaling pathway is one of the core pathways regulating cell fate in the whole life cycle of mammals and plays an important role in coordinating tissue and organ development. As the regulatory hub of the canonical Wnt signaling pathway, β-catenin is responsible for transmitting signals from the cytoplasm to the nucleus, triggering the transcription of downstream target genes, and initiating the Wnt signaling cascade.^30^ Without the Wnt signaling, β-catenin in the cytoplasm is bound and phosphorylated by its destruction complexes, including adenomatous polyposis coli, axis inhibition protein (Axin), glycogen synthase kinase 3β, and casein kinase 1α.^31^^,^^32^ Phosphorylated β-catenin subsequently undergoes ubiquitin-proteasomal degradation.^33^ When the Wnt ligands bind to its receptor (Frizzled and LRP), they induce the dissociation of β-catenin from the destruction complex in the cytoplasm.^31^^,^^32^ Unphosphorylated β-catenin accumulates in the cytoplasm and then translocates into the nucleus and activates the transcription of Wnt target genes.

The Wnt/β-catenin signaling pathway is indispensable in regulating embryonic development and tissue homeostasis. Dysregulation of the Wnt/β-catenin signaling pathway is associated with the occurrence of many different diseases, including cardiovascular,^34^^,^^35^ respiratory,^36^^,^^37^ and urinary diseases,^38^^,^^39^ and tumor development.^40^^,^^41^ Wnt/β-catenin signaling also controls the proliferation and differentiation of mesenchymal stem cells.^42^^,^^43^ It also plays a critical role in endochondral ossification and maintains skeletal tissue homeostasis.^42^^,^^43^ Abnormal Wnt/β-catenin signaling could lead to genetic and degenerative bone and joint diseases. OA is a multifactorial-involved and abnormal chondrocyte maturation-related degenerative joint disease. Recent evidence has shown that Wnt/β-catenin signaling plays a prominent role in the development and progression of OA.^44^, ^45^, ^46^ The analysis of susceptibility factors in OA patients showed that genes related to Wnt/β-catenin signaling were changed. Wnt7b was found to be significantly up-regulated in the cartilage and synovial of OA patients and was involved in the lesions of OA cartilage.^47^ The study also found that WISP-1, a Wnt-inducing signaling protein, was increased in human OA cartilage and synovium.^48^ These findings suggest that abnormal Wnt/β-catenin signaling is associated with OA cartilage lesions and synovial inflammation. Curl-associated protein 3 is an antagonist of Wnt signaling.^49^ Mutations in *FrzB*, the gene encoding curl-associated protein 3, are associated with primary hip OA and OA in other joints.^50^^,^^51^

**Figure 3:**
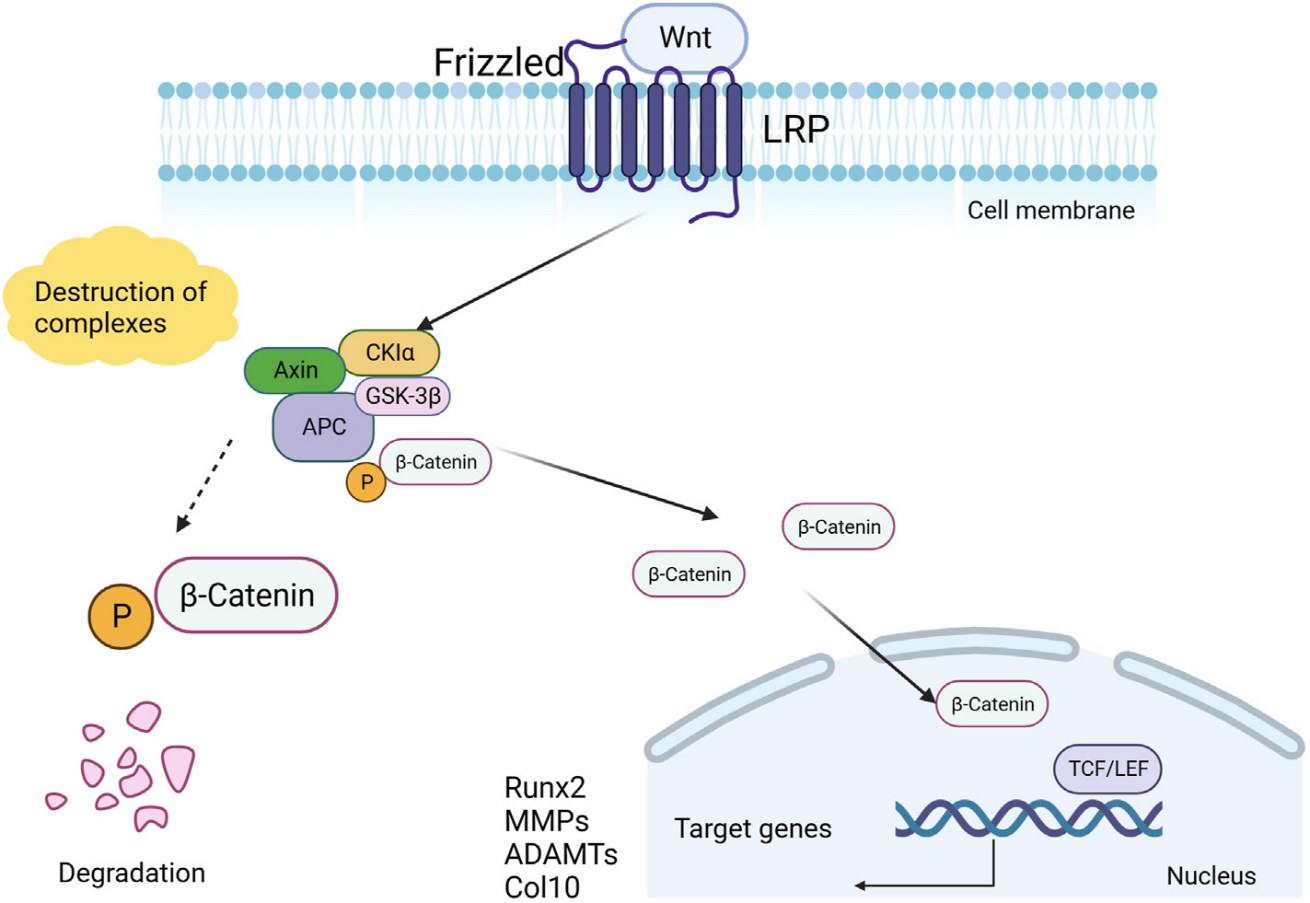
Activation and conduction of the Wnt/β-catenin signaling pathway in OA. Wnt/β-catenin signaling in healthy articular cartilage is tightly regulated. In the absence of Wnt binding to its ligand, β-catenin in the cytoplasm is phosphorylated by the destruction complex and is degraded by the proteasome after ubiquitination, and the Wnt/β-catenin signal is inhibited. In OA chondrocytes and synoviocytes, Wnt binds to its ligands Frizzled and LRP, resulting in the dissociation of the destruction complex in the cytoplasm, and β-catenin is freed from the destruction complex. β-catenin phosphorylation is reduced and β-catenin migrates into the nucleus. The Wnt/β-catenin signaling pathway activates the downstream target gene expression, including Runx2, MMPs, and ADAMTs, promoting the progression of OA. Axin, axis inhibition protein; CK1α, casein kinase 1α; GSK-3β, glycogen synthase kinase 3β; APC, adenomatous polyposis coli; Runx2, runt-associated transcription factor 2; MMPs, matrix metalloproteinase; ADAMTs, aggregate enzymes; Col10, collagen type X.

Altered expression of Wnt signaling-related genes was also observed in spontaneous OA mice and collagenase-induced OA mice. For example, WISP-1, Wnt-16, and Wnt-2B were up-regulated in these OA mouse models.^48^^,^^52^ Besides, the contribution of Wnt/β-catenin signaling in the development of OA was further investigated by generating β-catenin overexpression transgenic mice, and OA-like phenotypes were observed in multiple joints in the body in these mice, such as the knee joint, hip joint, temporomandibular joint, and facet joint of the spine.^53^, ^54^, ^55^, ^56^, ^57^ The tyrosine kinase Fyn aggravates the development of OA by promoting β-catenin phosphorylation and nuclear translocation.^58^ Inhibition of Fyn blocks the β-catenin pathway and reduces the levels of extracellular matrix catabolic enzymes in articular cartilage.^58^ In addition, inhibition of Wnt signaling in synovial tissue can reduce the expression of MMPs in synovial tissue.^59^^,^^60^ Axins (including Axin1 and Axin2) are crucial scaffold proteins in the β-catenin destruction complex, which play significant roles in the stability of β-catenin. Zhou et al generated *Axin1*^Agc1CreER^ mice to delete *Axin1* in aggrecan-expressing chondrocytes and found temporomandibular joint OA-like phenotype due to up-regulation of β-catenin signaling in these *Axin1* conditional knockout mice.^61^ The data showed that *Axin1* deficiency in condylar chondrocytes significantly increased the expression of β-catenin, MMP13, and ADAMT5.^61^ The loss of *Axin1* in chondrocytes leads to progressive OA-like changes in the temporomandibular joint, including vertical cracks on the cartilage surface and increased catabolic activity.^61^ Abnormal mechanical loading plays an important role in the degeneration of articular cartilage, and excessive activation of Wnt/β-catenin signaling caused by overloading can mediate degeneration of mandibular condyle cartilage.^62^ In addition, sclerostin is a typical Wnt inhibitor, and loss of its expression promotes arthritis progression in OA mice with joint instability.^63^ Studies have shown that increased mechanical loading inhibits the expression of sclerostin and activates Wnt/β-catenin signaling, ultimately promoting the progression of OA cartilage degeneration and advanced subchondral bone sclerosis.^64^^,^^65^ These findings have proved that the excessive transmission of Wnt/β-catenin signal mediates the occurrence and development of OA, and inhibition of β-catenin signaling may help to delay the progression of OA (Fig. 3).

### Runx2

Runx2 is a member of the Runt transcription factor family and plays a vital role in skeletal development at an embryonic stage and is involved in bone remodeling at postnatal and adult periods. Global deletion of *Runx2* in mice results in impaired chondrocyte and osteoblast differentiation leading to failure of the ossification process,^66^ suggesting that Runx2 plays a key role in chondrocyte and osteoblast differentiation. Research reports indicate that Runx2 is extensively expressed in pre-hypertrophic chondrocytes and regulates chondrocyte hypertrophy.^67^^,^^68^ Overexpression of *Runx2* in chondrocytes accelerates chondrocyte maturation and promotes endochondral ossification.^69^ Conversely, loss of Runx2 expression in chondrocytes inhibits chondrocyte hypertrophic differentiation and migration to the subchondral bone, thereby delaying endochondral ossification.^69^^,^^70^ Furthermore, studies found that Runx2 controls chondrocyte proliferation and cartilage growth by adjusting cell cycle genes such as cyclin A1.^71^ Runx2 is not only involved in the proliferation and differentiation of chondrocytes but also an important regulator of osteoblast differentiation. Heterozygous deletion of *Runx2* in humans leads to the appearance of cranial dysplasia manifested by open fontanelles and sutures, clavicle dysplasia, and short stature.^72^ Homozygous deletion of *Runx2* in mice causes a complete lack of bone formation causing the death of mice right after birth.^73^ In addition, Runx2 is required for bone matrix protein expression in osteoblasts and promotes the expression of major bone matrix protein genes such as osteopontin, integrin binding sialoprotein, osteocalcin, Col1a1, and Col1a2.^74^

Runx2 plays an important role in chondrocyte differentiation and maturation and maintenance of cartilage tissue homeostasis. Studies have shown that Runx2 is associated with the expression of matrix-degrading enzymes, suggesting that Runx2 overexpression appears to play a crucial role in the cartilage degeneration process.^75^ The study found that Runx2 expression significantly increased cartilage and synovial tissue in OA patients or OA animal models.^76^^,^^77^ Specific overexpression of *Ruxn2* in the articular chondrocytes accelerates the pathological progression of posttraumatic OA in adult mice.^78^ The conditional deletion of *Runx2* in chondrocytes effectively delays the evolvement of traumatic OA in adult mice.^75^ Articular cartilage degradation is the main pathological change of OA, and the matrix-degrading enzymes that mediate cartilage degradation include MMPs and aggrecanase enzymes (ADAMTs).^79^, ^80^, ^81^ MMPs and ADAMTs are zinc-dependent proteolytic enzymes that degrade collagen and aggrecan in the cartilage matrix, disrupting the integrity of joint cartilage. Existing studies support that MMP13 and ADAMTS5 are the targets of Runx2.^75^ *In vitro* cell experiments by Wang et al showed that overexpression of Runx2 in chondrocytes increased the responsiveness of fibroblast growth factor 2-induced up-regulation of MMP13 expression, while Runx2 knockdown by siRNA inhibited MMP13 expression.^82^ It has been widely recognized that excessive mechanical stress promotes the development of OA. Mechanical stress induction leads to up-regulation of MMP13 and ADAMTS5 expression, and Runx2 may be a key mediator for mechanical stress-induced MMP13 and ADAMTS5 expression.^83^ Overexpression of *Runx2* in chondrocytes leads to exacerbation of OA in mice, which may be related to the expression of Runx2-activated MMP13 and ADAMTS5.^78^ In addition, the decreased expression of MMP13 and ADAMTS5 was found in chondrocytes of *Runx2*-deficient mice.^75^ Interestingly, a recent study found that Runx2 exerts catabolic and anabolic effects under inflammatory conditions. Heterozygous knockout of *Runx2* in chondrocytes reduces MMP13 expression while inhibiting OA progression.^84^ In contrast, homozygous knockout of *Runx2* in chondrocytes inhibited Col2a1 protein levels and significantly accelerated the development of OA.^84^ In addition, Runx3 shows an opposite role to Runx2 in the development of OA. Under normal conditions, Runx3 protects articular cartilage by promoting the production of extracellular matrix proteins. At the same time, Runx3 knockout inhibits the expression of lubricin and proteoglycans and accelerates the development of surgery-induced OA.^84^ As a key downstream factor in multiple pathways, Runx2 responds to various biological signaling pathways and participates in the pathogenesis of OA.

### AMPK-β-catenin signaling interaction

As an important signaling factor related to the initiation and progression of OA, AMPK and β-catenin play different roles in the progression of OA. It has been known that AMPK-related metabolic disturbance of chondrocytes causes the occurrence of OA.^21^ It is well known that the Wnt/β-catenin signaling pathway is involved in the regulation of embryonic development and tissue homeostasis, and β-catenin, as the core factor of canonical Wnt signaling, is necessary for this signaling. Many studies have proven that the progression of OA is closely related to abnormal Wnt/β-catenin signaling in chondrocytes.^44^ However, there is no evidence to show the crosstalk between AMPK and β-catenin signaling pathways during the pathological process of OA until recently. A recent study showed that as the upstream signal of β-catenin, AMPK inhibits β-catenin^S552^ phosphorylation and prevents β-catenin nuclear translocation in chondrocytes.^85^

The proto-oncogene SRSF9 interacts with β-catenin messenger RNA to promote the expression of β-catenin and accumulate it in the cytoplasm, thus leading to the occurrence of disease.^86^ AMPK inhibits the interaction between SRSF9 and β-catenin by promoting the phosphorylation of SRSF9, ultimately inhibiting the synthesis of β-catenin protein.^86^ Glycogen synthase kinase 3β is a crucial regulator of β-catenin stability and its phosphorylation and activity are related to reactive oxygen species,^87^ and AMPK participates in the regulation of reactive oxygen species by regulating metabolic processes. Therefore, the regulation of AMPK signaling may also crosstalk with the β-catenin signaling pathway through the glycogen synthase kinase 3β pathway. These findings further support the interaction between AMPK and β-catenin signaling pathways. Furthermore, the interaction of the AMPK/β-catenin signaling pathway is associated with osteogenic differentiation of mesenchymal stem cells. Activation of the AMPK/β-catenin signaling pathway in mesenchymal stem cells promotes the bone morphogenetic protein 9-induced osteogenic differentiation.^88^ The crosstalk between AMPK and β-catenin suggests that these two signaling pathways may synergistically affect OA disease states. Activation of the AMPK signaling pathway in chondrocytes is known to have a protective effect on articular cartilage.^89^ A study by Zhu et al showed that AMPK activators can inhibit β-catenin signaling in chondrocytes, thereby exerting a protective effect on chondrocytes.^85^ Further studies of related mechanisms have found that the inhibitory effect of AMPK activators on β-catenin signaling is achieved by inhibiting phosphorylation and nuclear shift of β-catenin.^85^ Meanwhile, the activation of AMPK decreased the expression of β-catenin downstream target genes *Axin2* and cyclin D1.^85^ This study is the first exploration of signaling interactions between AMPK and β-catenin signaling pathways and further demonstrates that the interaction of different signaling pathways is crucial in the development of OA.

### β-catenin-Runx2 signaling interaction

**Figure 4:**
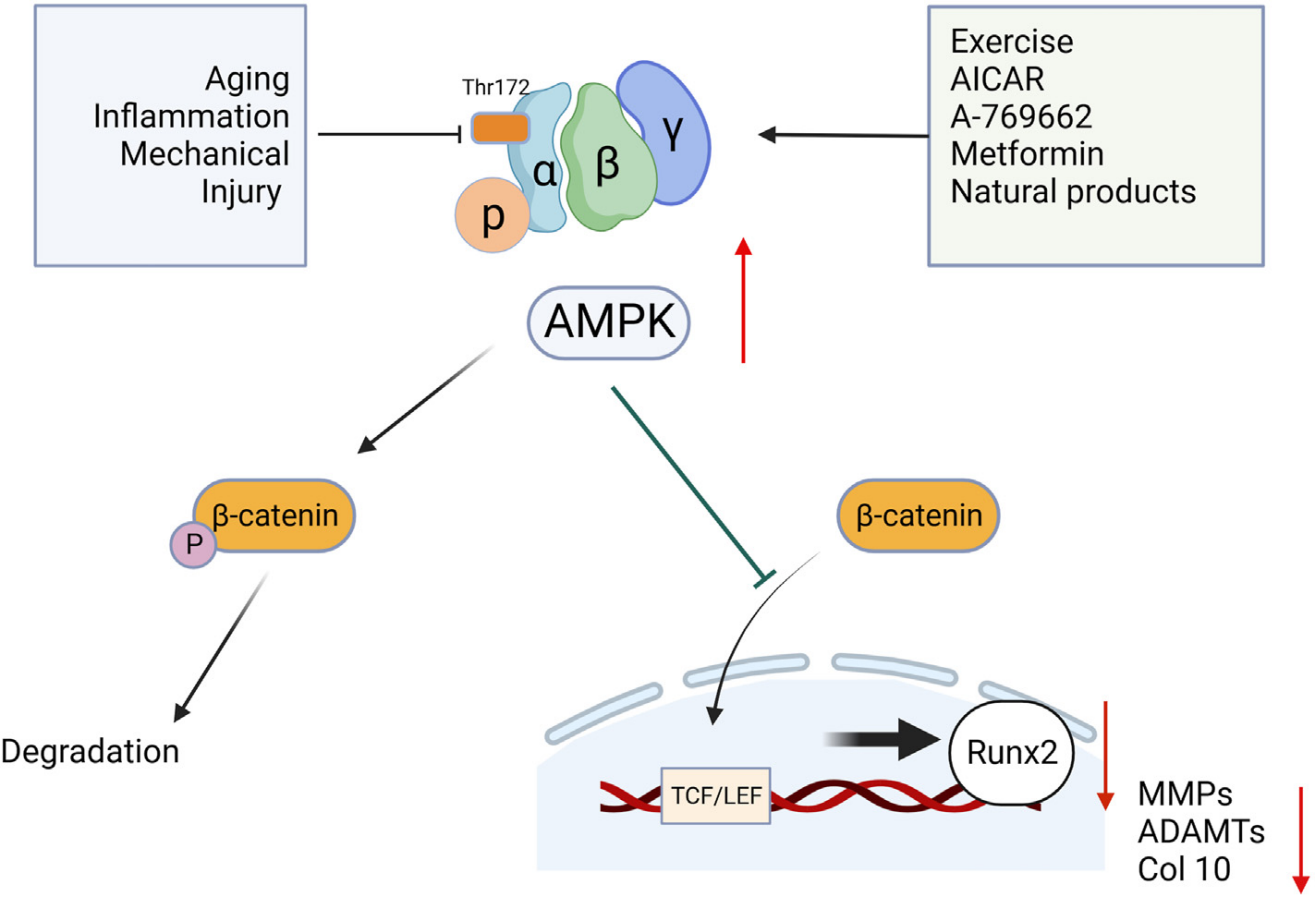
Conduction and crosstalk of AMPK-β-catenin-Runx2 signals in OA. AMPK, β-catenin, and Runx2 play unique roles in the disease development of OA, and the crosstalk between the three jointly regulates the pathological process of OA. AMPK acts as the upstream regulatory signal of β-catenin, and the conduction of AMPK signals can regulate the biological activity of β-catenin. Up-regulation of AMPK expression inhibits signaling of β-catenin by promoting its phosphorylation and degradation and blocking β-catenin nuclear translocation, ultimately inhibiting the downstream target gene Runx2 and reducing the expression of MMPs, ADAMTs, and Col-10. AICAR, 5-aminoimidazole-4-carboxamide 1-β-D-ribonucleoside; AMPK, AMP-activated protein kinase; Runx2, runt-associated transcription factor 2; MMPs, matrix metalloproteinase; ADAMTs, a disintegrin and metalloproteinase with thrombospondin motifs; Col10, collagen type X.

β-catenin is indispensable for tissue development and homeostasis, and Wnt/β-catenin signal transduction plays an important role in the transformation of physiological and pathological states of organisms. In the study of human genetic association, it was found that the mutations of the *FrzB* gene were associated with the susceptibility of patients to OA^50^ and have been further demonstrated in animal models.^90^^,^^91^ Runx2 is up-regulated in human and animal OA cartilage and has been considered the key event in OA articular cartilage degeneration.^76^ The idea that MMPs and ADAMTs are direct targets of Runx2 has been validated by numerous studies, and both are involved in cartilage degradation during OA.^92^ In addition, according to reports, Runx2 is an important downstream target of β-catenin, and the Wnt/β-catenin signal pathway can mediate chondrocyte hypertrophy by activating Runx2.^93^ Classical Wnt signaling regulates the expression of the *Runx2* gene *in vivo* to promote early bone development and maintain bone mass. The classical Wnt pathway in mesenchymal stem cells activates the expression of Runx2 via β-catenin/TCF1 and drives mesenchymal stem cell differentiation towards osteogenic lineages.^94^^,^^95^ More interestingly, β-catenin-mediated fluoride-induced aberrant osteogenesis is associated with up-regulation of Runx2 expression.^96^ Further, Wnt/β-catenin signaling mediates osteogenic differentiation and calcification of vascular smooth muscle cells by regulating the expression of the *Runx2* gene.^97^ Runx2 has been proven to be a downstream target gene of multiple pivotal signaling pathways such as Wnt/β-catenin, transforming growth factor-β/Smad, and nuclear factor-κB, and is involved in mediating the pathogenesis of hypertrophic chondrocyte differentiation. The role of Runx2 in the cartilage matrix degradation process in OA pathological state has been extensively reported.^77^ In general, β-catenin-Runx2 signaling plays an important role in both physiological and pathological processes. An overview of the interaction of β-catenin-Runx2 signaling in the pathological mechanism of OA will help to further understand the molecular pathway of OA and develop new therapeutic strategies (Fig. 4).

## Drug development aiming at targeting AMPK-β-catenin-Runx2 signaling

### AMPK signaling

AMPK maintains practical expression and phosphorylation in normal articular cartilage, and the activation and conduction of AMPK signaling in articular cartilage is an important factor for cartilage protection. The reduced expression of AMPK in the articular chondrocytes has been shown to be associated with the development of OA, and its mechanism of action in OA has been extensively studied.^4^^,^^21^^,^^98^^,^^99^ Significantly reduced AMPK phosphorylation was found in the cartilage of OA patients and joint tissues in various OA mouse models.^100^ This evidence has shown that abnormal AMPK activity is related to the pathogenesis of OA. In addition, AMPK is a key energy sensor that is integrated with the fundamental signaling network related to metabolic dysfunction and aging.^101^^,^^102^ Therefore, targeting AMPK will be an effective approach to the treatment of OA and other diseases (Table 1).

**Table 1:** Drugs or small molecules for the treatment of OA by targeting AMPK-β-catenin-Runx2 signaling.

Drugs or small molecules	Reference	Mechanism of action	Effect of treatment
AICAR, A-769662	Terkeltaub et al^22^ Zhao et al^23^ Husa et al^103^	Activates AMPK, up-regulates PGC-1α and Foxo3A, and down-regulates CHOP	Inhibits inflammatory response and oxidative stress damage and reduces cartilage catabolism
Protectin DX	Piao et al^105^	Activates AMPK and inhibits NF-κB	Reduces chondrocyte's inflammatory response and improves cartilage damage
l-carnitine	Liao et al^106^	Activates AMPK-ACC-CPT1	Reduces chondrocytes lipid accumulation and mitochondrial dysfunction and improves joint synovitis
17β-Estradiol	Mei et al^108^	Up-regulates SIRT1 to activate AMPK/mTOR	Promotes mitochondrial autophagy and protects chondrocytes
Metformin	Feng et al^89^ Zhu et al^85^ Wang et al^115^ Li et al^118^ Hyun et al^119^	Activates AMPK, down-regulates CGRP, and inhibits β-catenin	Regulates chondrocytes autophagy, reduces oxidative stress, inhibits inflammation, and improves pain
XAV-939	Lietman et al^122^	Inhibits Wnt/β-catenin	Inhibits chondrocyte catabolic reaction and improves cartilage and synovial inflammation
SAH-Bcl9, StAx-35R	Held et al^123^	Inhibits β-catenin	Inhibits chondrocyte hypertrophy, promotes matrix protein synthesis, and delays cartilage degradation
Salinomycin	Chen et al^127^	Down-regulates LRP6 and inhibits Wnt/β-catenin	Up-regulates chondrocyte anabolism and inhibits cellular catabolism and inflammation
Polydatin	Hu et al^128^	Inhibits Wnt/β-catenin	Inhibits inflammation, promotes matrix protein expression, and inhibits cartilage degradation
Erdosteine	Xi et al^126^	Inhibits Wnt/β-catenin	Inhibits chondrocyte inflammation, reduces extracellular matrix degradation, and slows down cartilage damage
SM04690	Deshmukh et al^134^	Inhibits Wnt/β-catenin	Inhibits cartilage inflammation and reduces joint cartilage destruction

Notes: AICAR, 5-aminoimidazole-4-carboxamide 1-β-D-ribonucleoside; AMPK, AMP-activated protein kinase; PGC-1α, peroxisome proliferator–activated receptor γ coactivator 1α; Foxo3A, forkhead box o3; CHOP, C/EBP homologous protein; NF-κB, nuclear factor-κB; CGRP, calcitonin gene-related peptide; LRP6, low-density lipoprotein receptor protein 6.

The *in vivo* metabolite of 5-aminoimidazole-4-carboxamide 1-β-D-ribonucleoside (AICAR) is an AMP mimic that non-selectively activates AMPK. In comparison, A-769662 is a selective AMPK activator. Both of them are pharmacological activators of AMPK, which can weaken the dephosphorylation of AMPK induced by interleukin-1β and TNF-α, and inhibit the pro-catabolic response to inflammatory factors in chondrocytes.^22^ The increased phosphorylation of AMPK induced by A-769662 is also involved in the regulation of chondrocyte catabolism and anti-oxidative stress by regulating the expression of forkhead box O3 and peroxisome proliferator-activated receptor γ coactivator 1α.^23^ Effective activation of AMPK can respond to biomechanical damage or interleukin-1β-induced responses by limiting the C/EBP homologous protein expression in chondrocytes.^103^ Protectin DX is a fatty acid metabolite with broad anti-inflammatory effects. It is related to AMPK signaling and can improve insulin resistance and inflammation in mice through the AMPK-dependent pathway.^104^ The latest research shows that protectin DX has a potential role in the treatment of OA.^105^ Protectin DX reduces the expression of chondrocyte inflammatory factors including nitric oxide, prostaglandin E2, inducible nitric oxide synthase, cycloxygenase-2, MMP3, MMP13, and ADAMTS4 through AMPK/nuclear factor-κB pathway.^105^ Further studies also found that protectin DX treatment significantly improved cartilage damage and reduced ORASI scores in OA rats.^105^ l-carnitine is a quaternary ammonium compound that regulates cellular energy metabolism and lipid metabolism. The study of Liao et al showed that l-carnitine reduces cellular lipid accumulation and mitochondrial dysfunction by regulating the AMPK-ACC-CPT1 signaling pathway and alleviates synovitis of knee OA.^106^

Autophagy is an important process in the regulation of cellular homeostasis that protects cartilage by regulating apoptosis and repairing the function of damaged chondrocytes. A decrease in autophagy activity has been observed at different stages of OA. AMPK has been shown to be associated with autophagy, promoting the autophagy process by regulating the expression of UNC-52-like kinase 1. Tang et al found that trehalose therapy restores oxidative stress-induced destruction of autophagic effect in mouse chondrocytes by activating the AMPK-UNC-52-like kinase 1 signaling pathway.^107^ The estrogen 17β-estradiol has been shown to mediate the AMPK/mTOR signaling pathway by promoting Sirt1 expression, improving chondrocyte mitophagy, and protecting chondrocytes, thereby potentially preventing the progression of OA.^108^

Metformin is the first-line drug for the treatment of diabetes and can activate AMPK signaling and affect metabolic and cellular processes. Metformin has been extensively studied for its therapeutic potential in a variety of diseases, including diabetes, neurodegenerative diseases, cardiovascular diseases, and tumors.^109^, ^110^, ^111^, ^112^ Recent research indicates that metformin shows great potential in the treatment of OA.^113^^,^^114^ Metformin attenuates cartilage damage and limits OA progression by activating the AMPK pathway to inhibit inflammation, regulate autophagy, and reduce oxidative stress.^89^^,^^115^ Besides, several recent researches have indicated that AMPK may be related to the inhibition of pain-related signaling, which is considered to be a potential new target for pain treatment.^116^ Reduced synthesis of neuronal transient receptor potential ankyrin 1 may be part of the molecular mechanism by which metformin inhibits pain.^117^ The study of the OA animal model found that metformin treatment can reduce the expression of pain-related mediator calcitonin gene-related peptide in the dorsal root ganglion, thereby reducing the pain sensitivity of OA animals.^118^^,^^119^ In general, metformin slows down the progression of OA by reducing cartilage inflammation, improving chondrocyte autophagy, and reducing the oxidative stress response. In addition, berberine, another AMPK activator, also inhibited surgically induced OA pathogenesis in mice.^120^

### β-catenin signaling

The role of β-catenin signaling in the occurrence of OA has been proven by many studies. It is well-recognized that β-catenin-mediated hyperactivation of canonical Wnt signaling is associated with OA development. Therefore, related molecules or drugs that target and regulate the β-catenin signaling pathway may provide a new strategy for developing disease-modifying OA drugs. XAV-939 is a small molecular inhibitor of Wnt/β-catenin, which stabilizes Axin2, thereby stabilizing the destruction complex and promoting phosphorylation and ubiquitination of β-catenin.^121^ Lietman et al showed that administration of XAV-939 by intra-articular injection inhibited Wnt/β-catenin signaling activation and improved OA progression in a traumatic OA mouse model.^122^ Further study found that XAV-939 can also suppress the immoderate proliferation of synovial fibroblasts, reduce the deposition of Col-1a1, and improve the inflammatory response of synovial tissue.^122^ StAx-35R and SAH-Bcl9 are two peptide mimics that inhibit β-catenin transcriptional activity and regulate Wnt/β-catenin signaling.^123^ *In vitro* experiments proved that StAx-35R and SAH-Bcl9 can block Wnt signal transduction in chondrocytes, promote the expression of SRY-box transcription factor 9 and aggrecan, inhibit the synthesis of Col-10a1, and prevent chondrocyte hypertrophy.^123^ Furthermore, a variety of other inhibitors targeting Wnt/β-catenin have been used in the study of OA therapy. Calcium channel blocker verapamil reduces Wnt/β-catenin signaling by enhancing *FrzB* promoter activity and inhibits the loss of chondrocyte proteoglycan, ameliorating OA progression *in vivo*.^124^ Treatment with polygalactic acid can slow down cartilage degeneration in rat OA models and reduce chondrocyte MMP expression and chondrocyte inflammation by inhibiting Wnt/β-catenin and MAPK signal pathways.^125^ The protective effects of metformin on articular cartilage mediated through the AMPK pathway have been demonstrated.^118^ Interestingly, studies by Zhu et al further suggest that metformin can slow the progression of OA by suppressing the transduction of the β-catenin signal.^85^ These data indicate that metformin protects cartilage through a range of mechanisms. Erdosteine, salinomycin, polydatin, fluoxetine, and strontium ranelate have also been shown to have the ability to inhibit Wnt/β-catenin signaling and may be potential drugs for OA treatment.^126^, ^127^, ^128^, ^129^, ^130^

Furthermore, the reports over the past few years have demonstrated that some microRNAs (miRNAs) play a critical role in the regulation of Wnt/β-catenin signaling. Intervening in the conduction of the Wnt/β-catenin signaling pathway by regulating the transcription of miRNAs has increasingly become a new strategy for the development of new OA therapeutics.^131^, ^132^, ^133^ The overactivation of β-catenin signaling has a crucial role in the initiation and progression of OA, and the development of small molecule inhibitors that suppress Wnt/β-catenin signaling will become a new approach for the development of disease-modifying OA drugs. SM04690 is a small molecule inhibitor that targets the Wnt signaling pathway. SM04690 can induce chondrocyte differentiation *in vitro* and inhibit the decomposition of inflammatory factor TNF-α in chondrocytes.^134^ *In vivo* experiments further demonstrated that SM04690 promoted articular cartilage growth and attenuated joint destruction in a rat OA model.^134^ The potential therapeutic value of SM04690 for OA has been recognized, and SM04690 is considered to be a promising disease-modifying OA drug. SM04690 could target Wnt/β-catenin signals and is currently in clinical trials. The evaluation of the phase I clinical trial showed that SM04690 improved pain scores and the width of joint space in OA treatment.^135^^,^^136^ The favorable OA therapeutic efficacy of SM04690 supports its role as a potential disease-modifying OA drug.^134^^,^^137^ The above studies suggest that inhibition of up-regulated Wnt/β-catenin signaling could maintain cartilage homeostasis and delay the development of OA cartilage damage. However, several issues need to be considered. i) Wnt/β-catenin signaling is also involved in the regulation of bone formation, which is increased during the remodeling and repair of subchondral bone in OA. Whether this process is related to the up-regulation of Wnt/β-catenin signaling requires more evidence. ii) It is not clear if inhibition of β-catenin signaling would be a valuable approach for OA treatment since it has the potential to inhibit bone formation. Intra-articular local administration of the anti-β-catenin approach may be an option. This requires further investigation.

### Runx2 signaling

The latest view suggests that Runx2 has an important role in the pathogenesis of OA and is associated with the expression of matrix-degrading enzymes in the articular chondrocytes.^77^^,^^81^ Up-regulation of Runx2 expression has been found in human and animal OA cartilage. Therefore, Runx2 is likely to be an attractive molecular target for future OA therapeutic studies. But so far, no drug or therapy targeting Runx2 has been developed for OA treatment. Since Runx2 is a transcription factor located in the nucleus, how to specifically target nuclear Runx2 expression and function in chondrocytes is the major concern in developing anti-Runx2 OA treatment.

miRNAs are a class of small endogenous noncoding RNAs that regulate gene transcription and expression by binding to the 3′-non-coding region of messenger RNAs.^138^ miRNA controls cell signal transduction by modulating the expression of a series of transcription factors and participates in the regulation of cellular physiological activities. Analysis of RNA-sequencing data in the serum and cartilage tissue revealed that miRNA expression patterns were altered in OA patients.^139^^,^^140^ miRNAs that can target Runx2 will be a potential tool to regulate Runx2.

miR-204-5p is a key miRNA regulating Runx2.^141^ Decreased levels of miR-204-5p were detected in human OA cartilage.^142^ Whereas increased expression of miR-204-5p altered Col-2a1, MMP13, and MMP1 expression in primary chondrocytes and SW-1 cells.^142^ Further *in vivo* experiments showed that miR-204-5p treatment could improve the OA-like phenotype in rats, mainly manifested by increased articular cartilage thickness and decreased Mankin score.^142^ These studies suggest that targeting miR-204-5p to regulate Runx2 expression may be an effective way to treat OA. miR-204 and miR-211 are two homologous miRNAs that regulate Runx2 expression in articular cartilage. A recent study showed that loss of miR-204/-211 expression, leading to abnormal accumulation of Runx2, ultimately leads to a severe and progressive OA phenotype, which includes cartilage destruction, synovial hyperplasia, subchondral sclerosis, and osteophyte formation.^143^ Intra-articular injection of adeno-associated virus overexpression of miR-204 can significantly down-regulate the expression of Runx2 in cartilage, reduce the formation of osteophytes in surgically induced OA model in mice, and alleviate joint synovial hyperplasia and cartilage degeneration.^143^ In addition, miR-105 was down-regulated in OA patients and negatively correlated with the expression of Runx2, ADAMTS7, and ADAMTS12 in cartilage.^138^ miRNA microarray analysis found that miR-105 is a key miRNA for the regulation of fibroblast growth factor 2, and fibroblast growth factor 2 is associated with OA cartilage destruction and vascular invasion.^144^ *In vitro* studies have shown that miR-105 is required for fibroblast growth factor 2/p65-mediated expression of Runx2, ADAMTS7, and ADAMTS12, and targeting miR-105 may be an ideal strategy for OA therapy.^138^ Other miRNAs that regulate Runx2 expression, such as miR-381 and miR-27b, have also been studied, providing more insights into the pathogenesis and therapeutic strategies of OA. In summary, the contribution of Runx2-targeting miRNAs to cartilage homeostasis and OA development is important, and the design of Runx2-targeted drugs for evaluating their therapeutic efficacy in OA treatment in animal models and humans will be one of the future research directions.

## Nature products in OA treatment

Effective OA treatment is important for patients with OA to improve their quality of life. At present, OA treatment includes drug therapy, physical therapy, and surgical intervention, aiming to relieve symptoms and reduce joint disability. However, there are no disease-modifying drugs that can change the structure damage of OA pathogenesis. Most drug treatments for OA mainly control pain symptoms, but cannot improve the pathological changes of diseased joint tissues such as articular cartilage, synovium, and subchondral bone. Therefore, there is an urgent need to find new potential therapeutic drugs for OA. Since some natural products have shown remarkable effectiveness in disease prevention and treatment, they have been extensively studied in the last decade and have received increasing attention. *In vitro* and *in vivo* studies over the past few years have reported that some natural products possess potent anti-inflammatory and anti-oxidant properties. Therefore, it is believed that natural extracts may also have the therapeutic potential for OA treatment. The therapeutic effect of natural ingredients derived from plant extracts on OA has been studied, and the underlying mechanisms have been revealed (Table 2).

**Table 2:** Natural products for the treatment of OA.

Natural products	Reference	Mechanism of action	Effect of treatment
Curcumin	Hartert et al^146^ Zhang et al^147^ Jin et al^149^ Guan et al^150^	Activates AMPK/PINK1/Parkin and inhibits NF-κB	Inhibits chondrocyte inflammation and catabolic reactions, improves mitochondrial autophagy, protects chondrocytes, and relieves pain
Resveratrol	Qin et al^152^ Wang et al^153^	Activates AMPK/mTOR, up-regulates hypoxia-inducible factor, and reduces NO and iNOS	Reduces inflammatory response and oxidative stress and promotes autophagy
Green tea catechin	Huang et al^155^^,^^156^	Inhibits mTOR and up-regulates Beclin1 and LC3	Promotes chondrocyte autophagy, inhibits apoptosis, and reduces cartilage degradation
Chlorogenic acid	Zada et al^157^	Activates NRF2 and NF-κB, inhibits Bcl-xL	Antagonizes oxidative stress damage, reduces chondrocyte apoptosis, and promotes autophagy
Quercetin	Qiu et al^160^ Feng et al^161^	Activates AMPK/SIRT1 and SIRT1/AMPK	Reduces endoplasmic reticulum stress and mitochondrial stress, weakens oxidative stress damage, and inhibits chondrocyte apoptosis
Icariin	Zeng et al^165^ Tang et al^166^	Inhibits NF-κB, PI3K/AKT/mTOR and inhibits β-catenin	Reduces chondrocyte's inflammatory response, regulates autophagy, and inhibits cartilage matrix degradation
Emodin	Ding et al^167^	Inhibits NF-κB and Wnt/β-catenin	Down-regulates the expression of matrix degradation enzymes to improve cartilage degradation
Berberine	Li et al^120^	Activates AMPK/SIRT1	Reduces inflammation, inhibits cartilage degradation, and relieves pain
Huzhangoside D	R.J. Zhang^168^	Inhibits AKT/mTOR and up-regulates beclin-1, ATG5, and ATG7	Inhibits chondrocyte apoptosis, promotes autophagy, and improves cartilage damage
Safflower yellow	Wang et al^169^	Activates AMPK/SIRT1 and inhibits NF-κB	Inhibits endoplasmic reticulum stress, reduces inflammatory response, and delays cartilage destruction

Notes: AMPK, AMP-activated protein kinase; NF-κB, nuclear factor-κB; NO, nitric oxide; iNOS, inducible nitric oxide synthase; mTOR, mammalian target of rapamycin; LC3, microtubule-associated protein light chain 3; NRF2, nuclear factor erythroid2-related factor 2; Bcl-xL, B-cell lymphoma-extra large; SIRT1, Sirtuin 1; PI3K, phosphoinositide 3-kinases; ATG5, autophagy-related protein 5; ATG7, autophagy-related protein 7.

Polyphenols are organic compounds that are found in most plants. Curcumin is a polyphenol extract from turmeric root, and its anti-inflammatory effects have been widely reported.^145^ The therapeutic efficacy of curcumin on OA has been extensively studied. *In vitro* experiments show that curcumin can effectively inhibit chondrocytes from producing inflammatory and catabolic mediators, such as IL-1, nitric oxide, and MMP3.^146^ In addition to slowing the progression of OA, curcumin was found to relieve pain symptoms in mice with post-traumatic OA.^147^ A recent meta-analysis showed that curcumin and turmeric extract can improve inflammation severity and pain sensitivity in individuals with arthritis.^148^ Several potential mechanisms may be involved in the therapeutic effect of curcumin on OA. In the study using OA models *in vivo* and *in vitro*, curcumin activates mitophagy through the AMPK/PINK1/PARKIN signaling pathway and exerts its protective effect on OA cartilage.^149^ Furthermore, the combined administration of curcumin and chondroitin sulfate was able to inhibit the nuclear factor-κB pathway and reduce cartilage damage and inflammatory responses in OA rats.^150^ Resveratrol is a plant polyphenol compound found in red grape skin. Resveratrol can inhibit the expression of nitric oxide and inducible nitric oxide synthase in chondrocytes stimulated by interleukin-1β and reduce oxidative stress damage in chondrocytes.^151^ Resveratrol promotes the expression of hypoxia-inducible factor-1α and -2α through the AMPK/mTOR signaling pathway, improves chondrocyte autophagy, and thereby slows down the degeneration of articular cartilage.^152^ In addition to its anti-inflammatory and anti-oxidant effects, resveratrol has also been reported to relieve OA pain. Wang et al found that resveratrol treatment could help alleviate the pain symptoms of monosodium iodoacetate-induced OA rats.^153^ The treatment of resveratrol on OA pain has also been partially proven in clinical trials. A randomized controlled study showed that the addition of resveratrol further increases meloxicam-mediated pain relief in patients with mild to moderate knee OA.^154^ Other polyphenolic compounds, such as green tea polyphenols^155^^,^^156^ and chlorogenic acid,^157^ also show great potential for the treatment of OA. In summary, polyphenols extracted from plants have extensive anti-inflammatory and anti-oxidant properties, so plant polyphenols may be developed as effective drugs for the treatment of OA.

Flavonoids and their derivatives play a potential role in OA treatment by improving inflammatory response in chondrocytes. Quercetin, a member of the flavonoid family, has possible therapeutic effects in malignancy, cardiovascular disease, and neurodegenerative diseases.^158^ Several research findings suggest that quercetin may have beneficial effects on endoplasmic reticulum stress and mitochondrial dysfunction.^159^ Quercetin attenuates oxidative stress-induced chondrocyte apoptosis through activation of the AMPK/SIRT1 signaling pathway, exerts chondro-protective effect, and prevents OA development.^160^^,^^161^ More interestingly, a recent study reported the role of quercetin in the regulation of gut microbiota and metabolome in the OA rat model.^162^ The idea that gut microbiota is associated with disease development has been widely proposed. Changes in gut microbiome composition and activity are associated with bone and joint diseases.^163^ Lan et al found that quercetin treatment significantly altered the fecal microbial profile of OA rats, including increased lactobacilli and decreased unidentified ruminococcaceae.^162^ Icariin is also a flavonoid compound, which is the main active ingredient of *S. shorthair*. Icariin has various pharmacological activities and is a potential anti-inflammatory drug.^164^ It has been reported that icariin treatment down-regulates MMP13 expression in chondrocytes treated with interleukin-1β, and its protective effect on chondrocytes is thought to be partially mediated by down-regulation of β-catenin activation.^165^ Tang et al reported that icariin alleviated OA by modulating autophagy in chondrocytes.^166^ These results show that flavonoid extracts have promising anti-OA therapeutic potential, which is of significance for the prevention and treatment of OA.

Other natural products such as avocado/soybean unsaponifiables, emodin, safflower yellow, huzhangoside D, coumarin, berberine, and many other natural active ingredients derived from plants have been shown to have considerable value in the treatment of OA.^120^^,^^167^, ^168^, ^169^, ^170^, ^171^ The potential clinical value and related mechanisms of plant natural ingredients have attracted broad attention.

## Summary

OA is a chronic degenerative disease affecting the entire joint, and its pathogenesis is complex and diverse, which has not been fully understood. AMPK, β-catenin, and Runx2 are closely related to the OA initiation and progression and are involved in the entire process of OA, including early- and late-stage OA development. The roles and mechanisms of these signaling molecules and their interactions in the development of OA have been extensively investigated in the past decade. Drugs targeting the AMPK-β-catenin-Runx2 signaling pathway have been explored in pre-clinical studies, and the potential therapeutic effects of these drugs for OA treatment have been demonstrated. In this review, we have summarized the latest findings in the research of roles and mechanisms of the AMPK-β-catenin-Runx2 signaling pathway in OA development, as well as the interactions between these molecules. At the same time, the progress of drug development targeting this signaling pathway for OA treatment is also discussed. Plant-derived natural products possess widespread anti-inflammatory and anti-oxidant properties and show great potential in the prevention and treatment of several diseases, including OA. We reviewed the present research on several natural products for their usage in OA treatment. However, further study is still required. For example, the mechanisms of action of natural products are complex and need to be carefully investigated. In addition, the efficacy of natural products, drug safety, and bioavailability still require further research. The effects of drug treatment are related to the doses and modes of action and the development of a more adequate drug delivery system will significantly improve drug usage. Although the development of a novel treatment for OA through targeting the AMPK-β-catenin-Runx2 signaling pathway holds great potential, more detail and in-depth studies are still required in the future.

## Conflict of interests

The authors declare that there are no competing interests.

## Funding

This work was supported by National Key Research and Development Program of China (No. 2021YFB3800800 to D.C. and L.T), the National Natural Science Foundation of China (No. 82030067, 82250710174, 82161160342, 82060406, 82360429 and 82172397 to D.C. and L.T. and Y.C.), the Hong Kong RGC (China) (No. HKU-17101821 to W.W.L and D.C.), Shenzhen Science and Technology Program (Guangdong, China) (No. JSGGKQTD20210831174330015 to H.P. and D.C.), and Natural Science Foundation of Guangxi (No. 2022JJA141126 to Y.C.) .
